# Continuous Subcutaneous Delivery of rhPTH(1-84) and rhPTH(1-34) by Pump in Adults With Hypoparathyroidism

**DOI:** 10.1210/jendso/bvae053

**Published:** 2024-03-29

**Authors:** Nipith Charoenngam, Erin Bove-Fenderson, Daniel Wong, Natalie E Cusano, Michael Mannstadt

**Affiliations:** Endocrine Unit, Massachusetts General Hospital, Harvard Medical School, Boston, MA 02114, USA; Endocrine Unit, Massachusetts General Hospital, Harvard Medical School, Boston, MA 02114, USA; Sutter Health, Sacramento, CA 95816, USA; Baylor Scott & White Dallas Diagnostic Association, Garland, TX 75044, USA; Department of Medicine, Division of Endocrinology, Lenox Hill Hospital, New York, NY 10022, USA; Endocrine Unit, Massachusetts General Hospital, Harvard Medical School, Boston, MA 02114, USA

**Keywords:** hypoparathyroidism, parathyroid hormone replacement therapy, recombinant human parathyroid hormone 1-84, recombinant human parathyroid hormone 1-34, teriparatide, PTH pump

## Abstract

**Context:**

Continuous subcutaneous infusion of recombinant parathyroid hormone (rhPTH) through a pump has been proposed as a therapeutic alternative for patients with chronic hypoparathyroidism who remain symptomatic or hypercalciuric on conventional treatment (calcium and active vitamin D) or daily injections of rhPTH(1-84) or rhPTH(1-34). However, the real-world evidence of the outcome of this novel therapy is limited.

**Case Descriptions:**

We report the clinical and biochemical outcomes of 12 adults with hypoparathyroidism (11 women, age 30-70 years, and 1 man, age 30 years) from 3 different clinical sites in the United States who were transitioned from conventional therapy to daily injections of rhPTH(1-84) or rhPTH(1-34) and then switched to continuous administration of rhPTH(1-84)/rhPTH(1-34) via pump therapy. In most patients, mean serum calcium concentrations increased while on PTH pump therapy compared with both conventional therapy (in 11 patients) and single/multiple daily rhPTH injections (in 8 patients). Despite this, 10 patients had lower median 24-hour urinary calcium levels while on PTH pump therapy compared with prior therapy (mean ± SD difference: −130 ± 222 mg/24 hours). All patients reported a qualitative decrease in hypocalcemic symptoms while receiving pump therapy. Three patients had pod failure at least once, and 1 patient developed an infusion site reaction.

**Conclusion:**

In this case series of 12 patients with chronic hypoparathyroidism treated with rhPTH(1-84)/rhPTH(1-34) administered via a pump, improvement in clinical and biochemical parameters were observed in the majority of the patients. Our observations indicate benefits of pump administration of rhPTH that warrant further investigation.

Hypoparathyroidism is a disease of inadequately low or absent circulating concentration of PTH that results in hypocalcemia and hyperphosphatemia [1]. This condition affects 23 to 37 per 100 000 individuals in the United States and Europe and is most commonly caused by removal of or accidental injury to the parathyroid glands during neck surgery and less commonly by genetic, idiopathic, and autoimmune etiologies [1, 2]. Clinical manifestations of hypoparathyroidism involve neuromuscular irritability associated with hypocalcemia, including muscle cramps, tingling, and seizures as well as neurocognitive and neuropsychiatric symptoms such as inability to focus and increased anxiety [3, 4].

Conventional treatment of hypoparathyroidism includes administration of a calcium supplement several times a day in addition to active vitamin D [4]. However, this treatment does not restore the physiologic actions of PTH, including renal reabsorption of calcium and renal excretion of phosphate. Therefore, hypercalciuria, hyperphosphatemia, and an elevated calcium-phosphate product are frequently observed. These biochemical adverse outcomes are associated with long-term complications, such as kidney stones and nephrocalcinosis [1-5].

In 2015, once-daily recombinant human (rh) PTH(1-84) was approved by the United States Food and Drug Administration (FDA) for the treatment of hypoparathyroidism [5, 6]. The product was recalled in 2019 due to technical problems with the delivery device that led to the potential for rubber particles to be found in the cartridge liquid [7]. Teriparatide [rhPTH(1-34)] has the same actions as endogenous PTH on calcium and phosphate homeostasis but has a relatively shorter duration of action than rhPTH(1-84) when administered subcutaneously [8]. A direct head-to-head comparison of the pharmacodynamics between the 2 forms of PTH has not been reported. Although it is not FDA approved for treatment of hypoparathyroidism in the United States, single and multiple daily subcutaneous administration of synthetic human PTH(1-34) has been shown to be effective in clinical trials for the treatment of postsurgical hypoparathyroidism [9-11] and other forms of hypoparathyroidism (ie, idiopathic, autoimmune polyglandular syndrome type 1, calcium-sensing receptor mutation) [12, 13]. Nevertheless, single or even multiple daily injections of the medications do not replicate continuous PTH secretion and, therefore, may not be consistent in correcting biochemical abnormalities and alleviating symptoms for all hypoparathyroid patients [13-15]. This limitation is due in part to the relatively short pharmacokinetics of subcutaneously administered rhPTH(1-84) (half-life 2.5-3 hours) [16] and rhPTH(1-34) (approximate half-life 1 hour) [8]. Furthermore, individualized dosing and adjustments during the day, for example when engaging in physical exercise, might be more easily accomplished through pump therapy.

In cases in which single or multiple daily injections of rhPTH(1-84) or rhPTH(1-34) are insufficient for controlling symptoms and biochemical abnormalities of hypoparathyroidism, continuous subcutaneous infusion via insulin pump may be considered as a therapeutic alternative. While the use of pump therapy with human PTH(1-34) [hPTH(1-34)] is not FDA approved, it has been shown to be effective for treatment of hypoparathyroidism in case reports [17-21] and clinical trials [22, 23]. On the other hand, the effects of pump therapy with rhPTH(1-84) in patients with hypoparathyroidism have not been reported, and the real-world evidence of the outcome of this novel mode of administration outside of clinical trials is relatively limited. Therefore, the objective of this case series is to demonstrate the clinical and biochemical outcomes of adults with chronic hypoparathyroidism who were transitioned from conventional therapy to subcutaneous injections of rhPTH(1-84) or rhPTH(1-34) and then switched to continuous administration of rhPTH(1-84) or rhPTH(1-34) via pump therapy.

## Methods

### Patients

We reviewed medical records of adult patients with chronic hypoparathyroidism who received continuous subcutaneous delivery of rhPTH(1-84) or rhPTH(1-34) through a pump from 2015 through 2022 at 3 different endocrine outpatient clinics in the United States, including the Massachusetts General Hospital (Massachusetts), Sutter Medical Center (California), and Lenox Hill Hospital (New York). All patients were transitioned from conventional therapy with oral calcium and calcitriol to subcutaneous injections of rhPTH(1-84)/rhPTH(1-34) and then switched to continuous subcutaneous administration of rhPTH(1-84)/rhPTH(1-34) using the Omnipod insulin pump. The diagnosis of hypoparathyroidism was confirmed by the treating physician based on the patient's clinical and biochemical status. The treatment was initiated at each site as part of routine clinical care. The retrospective review of the cases for this publication was approved by the Mass General Brigham Institutional Review Board (2020P000039).

### Pump Therapy

During pump delivery, the Omnipod insulin pump device (Insulet Corporation) was filled with rhPTH(1-84) or rhPTH(1-34) diluted in sterile normal saline. The pod was attached to the lower back, upper arm, or abdomen and changed approximately every 72 hours. Basal rates were programmed into a wireless device that controlled the pump delivery rate. Initial basal rates of administration were estimated to result in total daily doses that would be equivalent to approximately 80% of the previous rhPTH(1-84)/rhPTH(1-34) dose requirements of single/multiple daily injections. For example, for a patient who was being transitioned to rhPTH(1-84) pump therapy and was on 50 µg per day of rhPTH(1-84) given as multiple daily injections, pump therapy would be initiated at approximately 40 µg per day. In the case of this patient, 21 units (210 µL) of rhPTH(1-84) from a 50 µg-rhPTH(1-84) cartridge (equivalent to ∼150 µg) would be diluted in 129 units (=1290 µL) normal saline to obtain 150 µg of rhPTH diluted in 150 units (1500 µL) of solution. Then, the pump would administer the medication at the rate of 1.7 diluted units per hour, which equals 40.8 µg of rhPTH(1-84) per 24 hours. While the pod was changed every 3 days, each pod's content could last for longer (for a total of approximately 3.6 days for the previous example). The infusion rates were titrated at each outpatient visit based on symptoms, serum calcium concentrations, and 24-hour urinary calcium levels. Of note, the stability of rhPTH(1-84) or rhPTH(1-34) mixed with normal saline has not been validated. However, the use of diluted rhPTH(1-34) in sterile water for pump therapy has been reported [17].

### Study Measurements and Data Analysis

For each patient, we recorded the following clinical and biochemical parameters: daily doses of oral calcium supplements, calcitriol, and hydrochlorothiazide; daily doses and frequency of rhPTH(1-84)/rhPTH(1-34); emergency department visits; all available data with dates on albumin-corrected serum calcium, phosphate, magnesium, and creatinine concentrations; all available data with dates on 24-hour urinary calcium; and symptoms data and overall well-being recorded by chart review. Symptoms associated with hypocalcemia (ie, tingling, perioral numbness, muscle cramps, seizure, neurocognitive symptoms) and hypercalcemia (ie, constipation, fatigue, polyuria, neurocognitive symptoms) were recorded qualitatively. Biochemical data were analyzed descriptively and reported as scatter and box plots, mean ± SD, time-weighted averages ± time-weighted SD, and time-weighted proportion of serum calcium concentration in the range of <8.5, 8.5 to 10, and >10 mg/dL. These cutoffs were used in this report based on the typical lower limit of laboratory-normal serum calcium concentration of 8.5 mg/dL and because a serum calcium concentration of >10 mg/dL is likely to lead to increased urinary calcium. Of note, if a patient had multiple consecutive serum calcium concentrations in the event of a hospital admission for hypocalcemia or hypercalcemia, only the mean value of the first 2 serum calcium concentrations would be shown in the scatter plots, since the subsequent values would not represent the efficacy of the outpatient therapy. For statistical analysis, the paired *t*-test was employed to compare serum calcium and 24-hour urinary calcium data between different treatment periods (PTH pump therapy vs prior therapy), while the independent samples *t*-test was utilized to compare serum and urinary calcium data between patients receiving rhPTH(1-84) pump therapy and those receiving rhPTH(1-34) pump therapy. Normality of the data was verified using the Kolmogorov–Smirnov test. Statistical significance was defined as *P*-value <.05. Data illustrations and statistical analysis were generated using the GraphPad Prism software 9.4.0 (GraphPad, La Jolla, CA, USA).

## Results

A total of 13 adult patients with chronic hypoparathyroidism were initially identified from the medical records. One patient was excluded from the study due to the lack of available 24-hour urinary calcium level data. Finally, 12 adult patients with chronic hypoparathyroidism (11 women, age 30-70 years, and 1 man, age 30 years) who received continuous delivery of rhPTH(1-84) or rhPTH(1-34) were included in this analysis (Table 1). Eleven patients had chronic postsurgical hypoparathyroidism, and 1 patient had idiopathic hypoparathyroidism. Three patients (nos. 9, 11, and 12) had a history of kidney stones, and 1 patient (no. 11) had nephrocalcinosis. Four patients (nos. 4, 5, 7, and 10) had no evidence of kidney stones or nephrocalcinosis by ultrasound. The remaining patients did not have available renal imaging studies. All 12 patients were initially transitioned from conventional therapy to once-daily rhPTH(1-84) injection. All but 1 patient (no. 8) were then transitioned to multiple daily injections of rhPTH(1-84). Because rhPTH(1-84) was recalled by the manufacturer in September 2019, 1 patient (no. 8) was switched from once-daily injection of rhPTH(1-84) to multiple daily injections of rhPTH(1-34), and 3 patients (nos. 9, 10, and 12) were switched from multiple daily injections of rhPTH(1-84) to rhPTH(1-34). All patients were transitioned from multiple daily injections to pump therapy because they remained symptomatic despite acceptable serum calcium concentrations at the time they were checked. Five patients (nos. 1, 2, 3, 4, and 7) received pump-administered rhPTH(1–84), 6 patients (nos. 6, 8, 9, 10, 11, and 12) received pump-administered rhPTH(1-34), and 1 patient (no. 7) initially received pump-administered rhPTH(1-84) and was later switched to rhPTH(1-34). One patient (no. 7) had calcium supplements that were stopped while on daily injections of rhPTH(1-84) but were restarted at 1000 mg/day while on the pump due to the patient's preference. For all other patients, doses of calcium supplements were decreased or remained unchanged while on pump therapy compared to when they were on multiple daily injections. The mean ± SD decrease in the doses of calcium supplements while on pump therapy compared to their highest doses on prior treatment was 1329 ± 1028 mg/day (range: 100-3600 mg/day).

**Table 1.** Patient demographics and treatment summary

no.	Age, sex (years, M/F)	Race, ethnicity	Cause of HypoPTH	Treatment regimen										
				Pre-PTH		PTH QD			PTH BID/TID			PTH pump		
				Ca^2^^+^ (mg/d)/ VD (mcg/d)/ HCTZ (mg/d)	Duration (Years)	Ca^2^^+^ (mg/d)/ VD (mcg/d)/ HCTZ (mg/d)	rhPTH(1–84) (mcg/day)	Duration (Years)	Ca^2^^+^ (mg/d)/ VD (mcg/d)/ HCTZ (mg/d)	rhPTH(1–84/1-34) (mcg/day)	Duration (Years)	Ca^2^^+^ (mg/d)/ VD (mcg/d)/ HCTZ (mg/d)	rhPTH(1–84/1-34) (mcg/day)	Duration (Years)
1	65 F	W/NH	PS/ThCa	NR/0.25/0	5.1	750/0/0	rhPTH (1-84): 50.0 ± 0.0	5.1	375/0/0	rhPTH (1-84): 49.4 ± 2.4	2.0	0/0/0	rhPTH (1-84): 54.2 ± 1.8	4.8
2	70 F	W/NH	PS/ThCa	1900/0.38	3.3	1000/0.25/0	rhPTH (1-84): 47.9 ± 14.5	3.1	1000/0.25	rhPTH (1-84): 68.6 ± 3.8	1.9	500/0	rhPTH (1-84): 60.6 ± 1.1	5.0
3	50 F	W/NH	PS/ThCa	1000/0.25/0	13.8	693/0/0	rhPTH (1-84): 70.7 ± 8.8	2.9	0/NR/0	rhPTH (1-84): 95.7 ± 10.3	2.0	0/NR/0	rhPTH (1-84): 44.9 ± 0.7	1.8
4	50 F	W/NH	PS/ThCa	NR/NR/0	2.2	0/0/0	rhPTH (1-84): 100.0 ± 0.0	8.8	0/0/0	rhPTH(1-84): 82.1 ± 8.6	4.0	0/0/0	rhPTH (1-84): 64.3 ± 1.0	1.4
5	30 F	W/NH	PS/goiter with compressive symptoms	3600/2/0	1.2	0/0/50	rhPTH (1-84): 77.9 ± 24.9	1.0	0/0/50	rhPTH (1-84): 52.6 ± 16.9	1.7	0/0/0	rhPTH (1-84): 57.1 ± 9.5	0.2
6	59 F	W/NH	PS/ThCa	NR/0.38/12.5	2.5	NR/0/12.5	rhPTH (1-84): 50.0 ± 0.0	0.5	600/0/12.5	rhPTH (1-84): 50.0 ± 0.0	2.2	500/0/12.5	rhPTH(1-84): 40.8 ± 0.0	4.4
7	50 F	W/NH	PS/MNG	1250/0.75/0	6.7	0/0.25/0	rhPTH (1-84): 98.9 ± 7.2	4.3	0/NR/0	rhPTH (1-84): 84.0 ± 0.0	0.7	1000/0/0	rhPTH (1-84): 87.6 ± 3.0 rhPTH(1-34): 20.0 ± 0.0	6.7
8	59 F	W/NH	PS/ThCa	3000/0.50/25	11.3	1200/0/0	rhPTH(1-84): 100.0 ± 0.0	4.5	0/0/0	rhPTH(1-34): 37.5 ± 0.0	0.3	0/0/0	rhPTH(1-34): 30.0 ± 0.0	2.4
9	51 F	W/NH	PS/ThCa	1200/0.50/25	4.1	0/0/25	rhPTH(1-84): 55.6 ± 10.4	0.6	0/0/25	rhPTH(1-84): 50.5 ± 18.0 rhPTH(1-34): 12.7 ± 1.3	4.5	0/0/0	rhPTH(1-34): 17.6 ± 2.6	1.8
10	59 F	W/NH	PS/ThCa	1500/0.50/0	1.5	250/0/0	rhPTH(1-84): 50.0 ± 0.0	0.2	0/0/0	rhPTH(1-84): 50.0 ± 0.0 rhPTH(1-34): 15.0 ± 0.0	2.3	0/0/0	rhPTH(1-34): 20.5 ± 4.3	1.3
11	30 M	W/NH	Idiopathic	1200/0.5/25	12.9	0/0/25	rhPTH(1-84): 50.0 ± 0.0	0.6	0/0/25	rhPTH(1-84): 50.0 ± 0.0	1.9	0/0/0	rhPTH(1-34): 16.0 ± 0.0	2.6
12	53 F	AI/AN/NH	PS/thyroid nodule and HT	NR/0.38/0	5.0	1000/0.38*^a^*/0	rhPTH(1-84): 50.0 ± 0.0	0.8	500/0.5/0	rhPTH(1-84): 26.5 ± 6.5 rhPTH(1-34): 10.0 ± 0.0	4.4	375/0/0	rhPTH(1-34): 8.1 ± 0.2	2.2

Pairwise comparisons between treatment periods were performed using the paired *t*-test. Ca^2+^/VD/HCTZ values are the median of all reported doses over the period indicated by treatment regimen. rhPTH (1–84)/rhPTH (1-34) values are the time-weighted mean ± SD of all reported doses over the period indicated by treatment regimen.

Abbreviations: AI/AN, American Indian or Alaskan Native; BID/TID, multiple daily parathyroid hormone injections; Ca^2+^, serum calcium; F, female; H, Hispanic; HCTZ, hydrochlorothiazide; HT, Hashimoto thyroiditis; HypoPTH, hypoparathyroidism; M, male; MNG, multinodular goiter; NH, non-Hispanic; NR, not reported; Pre-PTH, conventional therapy (calcium and active vitamin D); pump, parathyroid hormone pump therapy; PS, postsurgical; QD, single daily parathyroid hormone injection; rhPTH(1-34), recombinant human parathyroid hormone 1–34; rhPTH(1–84), recombinant human parathyroid hormone 1–84; ThCa, thyroid cancer; VD, calcitriol; W, White.

^*a*^Patient no. 12 was on alternate-day dosing of 0.25 to 0.5 mcg/day of calcitriol during conventional therapy.

.

In addition, all but 1 patient (no. 7) took calcium supplements of not more than 500 mg/day, and none of the patients reported taking calcitriol while on pump therapy. Three patients (nos. 5, 9, and 11), who were on hydrochlorothiazide while receiving single and multiple daily PTH injections, stopped the medication while on pump therapy. The means ± SD of duration of treatment with conventional therapy, once-daily injection, multiple daily injection, and PTH pump therapy were 5.8 ± 4.3, 2.7 ± 2.5, 2.3 ± 1.3, and 2.9 ± 1.8 years, respectively. Among the 7 patients (nos. 1-7) who transitioned from multiple daily injections of rhPTH(1-84) to rhPTH(1-84) pump therapy, 4 patients (nos. 2, 3, 4, and 6) exhibited a lower time-weight average in the rhPTH(1-84) dose. Among the 4 patients (nos. 8, 9, 10, and 12) who transitioned from multiple daily injections of rhPTH(1-34) to rhPTH(1-34) pump therapy, 2 patients (nos. 8 and 12) had a lower time-weight average in the rhPTH(1-34) dose (Table 1). The mean ± SD differences in the time-weight average doses of rhPTH(1-84) (for patients 1-7) and rhPTH(1-34) (for patients 8, 9, 10, and 12) while on pump therapy compared with multiple daily injections were −10.4 ± 19.8 mcg/day and 0.3 ± 6.2 mcg/day, respectively. Variations of PTH doses [calculated by 100 × (max PTH dose—min PTH dose)/max PTH dose] after 3 months of initial titration while on PTH pump therapy varied from 0% to 32% (Supplementary Table S1) [24].

**Figure 1.**
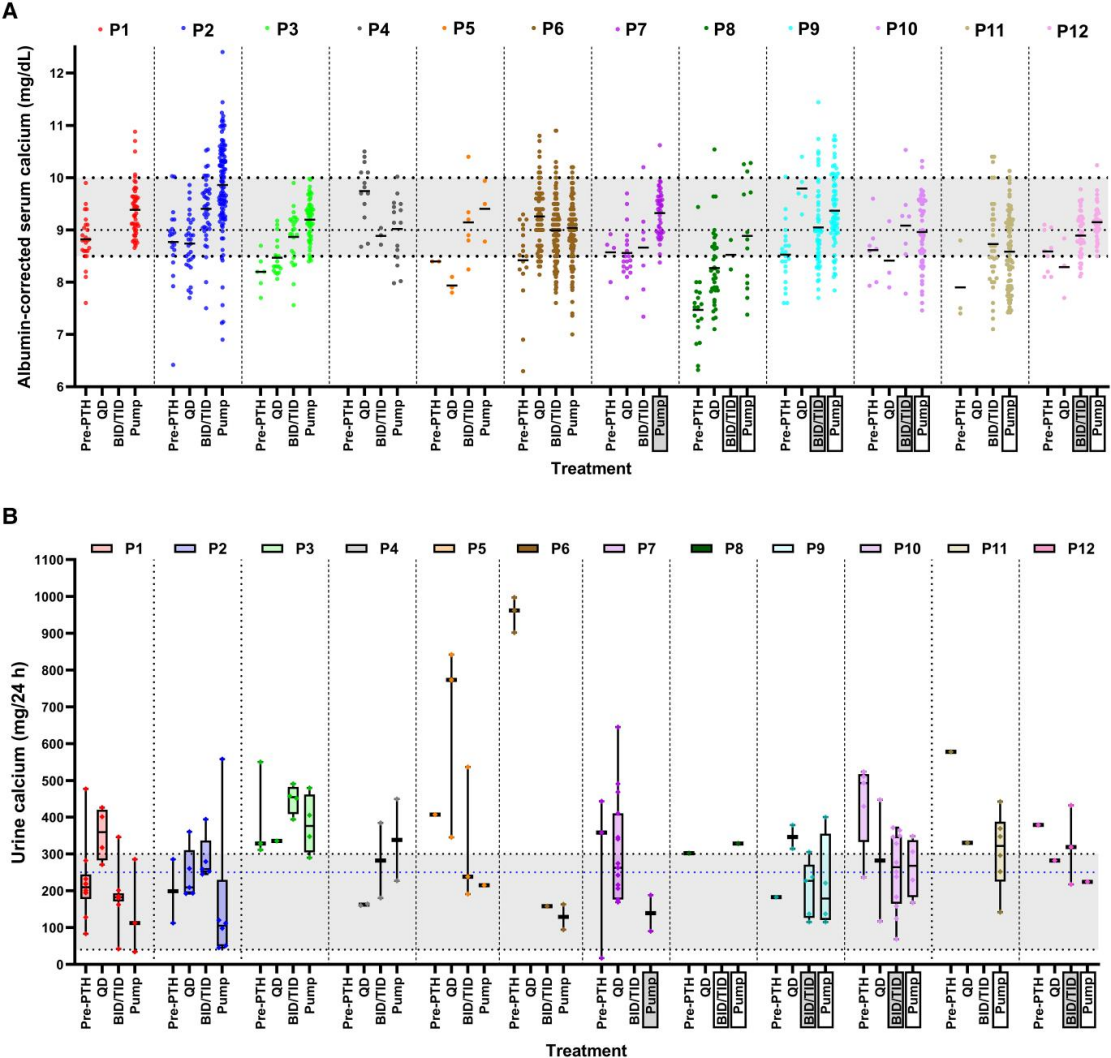
Serum calcium (A) and 24-hour urine calcium (B) during different treatment periods. The white box indicates treatment with recombinant human parathyroid hormone 1-34. The gray box indicates treatment with recombinant human parathyroid 1-84 that was later transitioned to recombinant human parathyroid hormone 1-34. The rest are treatment with recombinant human parathyroid 1-84. Abbreviations: BID/TID, multiple daily parathyroid hormone injections; P, patient; Pre-PTH, conventional therapy (calcium and active vitamin D); pump, parathyroid hormone pump therapy; QD, single daily parathyroid hormone injection.

As shown in Fig. 1, serum calcium tended to increase while on PTH pump compared with prior treatment in many patients. Despite this, their 24-hour urinary calcium decreased (nos. 1, 2, 5, 6, 7, 9, 10, and 12). Eleven patients had higher mean serum calcium concentrations while on PTH pump therapy than when they were on conventional therapy (calcium and active vitamin D, Fig. 1A). One patient (no. 4) did not have available serum calcium concentrations while on conventional therapy. Eight patients (nos. 1, 2, 3, 5, 7, 8, and 12) had higher mean serum calcium concentrations while on PTH pump therapy than when they were on single/multiple daily PTH injections (Fig. 1A).

As shown in Fig. 1B, all patients had at least 1 elevated 24-hour urinary calcium (defined as >300 mg/24 hours for men, > 250 mg/24 hours for women [4]) when they were on conventional therapy or single/multiple daily PTH injections. The frequency of high urinary calcium ranged from 25% to 100% of the measurements. Ten patients (nos. 1, 2, 3, 5, 6, 7, 9, 10, 11, and 12) had lower median 24-hour urinary calcium levels during PTH pump therapy compared to the median levels during prior therapy. The mean ± SD of patient-median 24-hour urinary calcium levels during PTH pump therapy compared with prior therapy was 228 ± 93 vs 358 ± 192 mg/24 hours. The difference was not statistically significant (mean difference: −130 ± 222 mg/24 hours, *P* = .078). Among them, 5 patients (nos. 1, 5, 6, 7, and 12) achieved all 24-hour urinary calcium within the normal range on PTH pump therapy.

**Figure 2.**
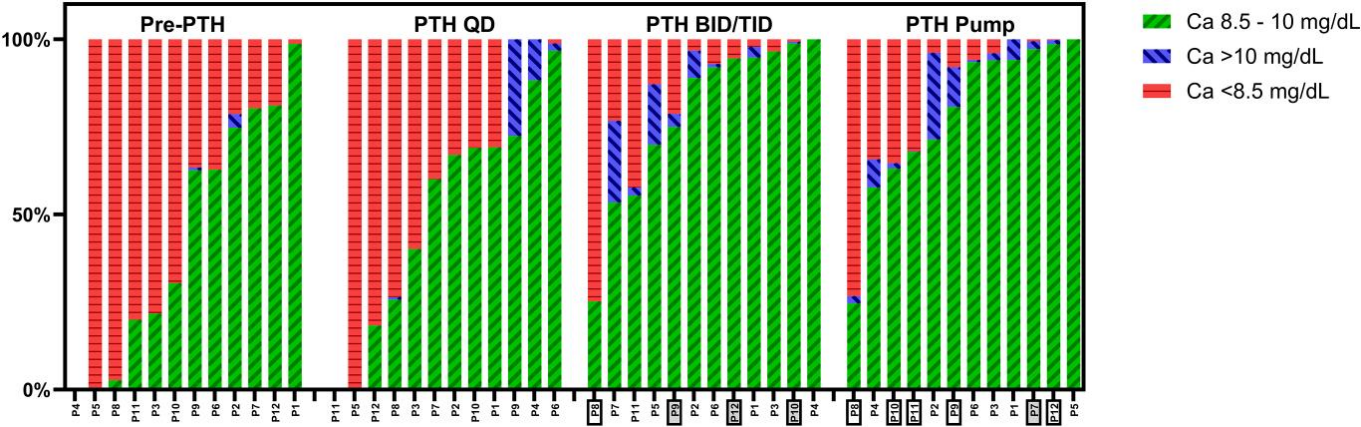
Time-weighted percentage of different categories of albumin-corrected serum calcium concentrations during different treatment periods. The white box indicates treatment with recombinant human parathyroid hormone 1-34. The gray box indicates treatment with recombinant human parathyroid 1-84 that was later transitioned to recombinant human parathyroid hormone 1-34. The rest are treatment with recombinant human parathyroid 1-84. Abbreviations: BID/TID, multiple daily parathyroid hormone injections; Ca, calcium; P, patient; Pre-PTH, conventional therapy (calcium and active vitamin D); pump, parathyroid hormone pump therapy; QD, single daily parathyroid hormone injection.

**Figure 3.**
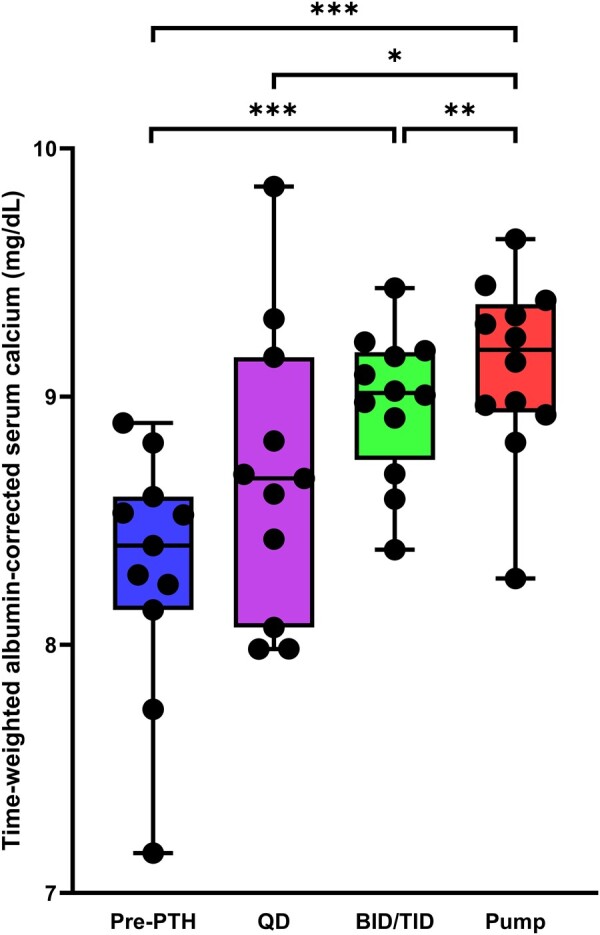
Time-weighted mean and SD of serum calcium concentration during different treatment periods. *, **, *** denote *P* < .05, *P* < .01, and *P* < .001, respectively.

As shown in Fig. 2, the proportion of time with calcium in the range of 8.5 to 10 mg/dL was numerically higher while on pump therapy and multiple daily injections compared with conventional therapy and single daily injection. The mean patient-time-weight average of serum calcium concentration on PTH pump therapy was statistically significantly higher on PTH pump therapy compared with conventional therapy, single daily injections, and multiple daily injections (all *P* < .05) (Fig. 3). The time-weighted averages of serum calcium concentration on PTH pump therapy were higher compared with conventional therapy in all but 1 patient (no. 4, with unavailable data on conventional therapy) and were higher compared with single and/or multiple daily PTH injections in 8 patients (nos. 1, 2, 3, 5, 7, 10, 11, and 12; Fig. 3, Supplementary Fig. S1) [24]. The mean ± SD differences for patient-time-weight averages of serum calcium on PTH pump therapy compared with conventional treatment was 0.8 ± 0.2 mg/dL. The mean ± SD differences were less pronounced when comparing PTH pump therapy with single daily PTH injections (0.5 ± 0.6 mg/dL) and multiple daily PTH injections (0.1 ± 0.1 mg/dL).

No statistical significant differences in serum calcium concentrations and 24-hour urinary calcium levels were observed between patients receiving rhPTH(1-84) pump (nos. 1-6, mean ± SD patient-time-weight average serum calcium 9.3 ± 0.2 mg/dL, mean ± SD patient-median urinary calcium 212 ± 95 mg/24 hours) and those receiving rhPTH(1-34) pump (nos. 8-12, mean ± SD patient-time-weight average serum calcium 8.9 ± 0.4 mg/dL, mean ± SD patient-median urinary calcium 264 ± 64 mg/24 hours). Of note, significant fluctuations in serum calcium concentrations were observed in certain patients. For example, patients 2 and 9 experienced multiple episodes of hypercalcemia during the follow-up period as they reported symptoms such as leg cramps and fatigue even at normal serum calcium concentrations. However, it was unclear whether these symptoms were directly associated with their serum calcium concentrations.

While the number of measurements of serum phosphorous, magnesium, and creatinine were limited, the means of serum phosphorous concentrations were lower on PTH pump therapy than conventional therapy in 8 patients (nos. 1, 2, 3, 5, 7, 8, 9, and 10) (Supplementary Fig. S2, Supplementary Table S2) [24]. Two patients (nos. 4 and 6) remained hyperphosphatemic while on PTH pump therapy. Mean serum magnesium concentrations were in the normal or high normal range in all patients while on pump therapy and higher than conventional therapy or once daily PTH in 6 patients. The mean ± SD differences for patient-average serum phosphorus and serum magnesium on PTH pump therapy compared with conventional treatment were −0.9 ± 1.0 mg/dL and 0.1 ± 0.3 mg/dL, respectively. Serum creatinine concentrations were relatively stable in all patients. The frequency of measurements of biochemical data during different treatment periods is summarized in Supplementary Table S3 [24].

All of the patients reported a qualitative decrease in symptoms while receiving pump therapy, including a lower incidence of fatigue, brain fog, tingling, and muscle cramps compared to standard therapy and daily injections. One patient (no. 6) reported self-limited irritation of the tongue while receiving rhPTH(1-34). One patient (no. 2) reported a skin infection at the infusion site, requiring a change of the infusion site. One patient (no. 11) had 1 pod failure, and 2 patients (nos. 4 and 6) had multiple pod failures. One patient (no. 5) had 6 emergency department visits due to hypocalcemia while on single/multiple daily PTH injections and had only 1 emergency department visit while on PTH pump therapy of similar duration. Another patient (no. 12) had 2 emergency department visits for hypocalcemia while on multiple daily PTH injections and had no emergency department visits while on PTH pump therapy of similar duration. Other patients did not report any emergency department visit for hypo- or hypercalcemia.

## Discussion

In this case series, we retrospectively report clinical and biochemical outcomes of 12 adult patients with chronic hypoparathyroidism who were treated with long-term continuously infused subcutaneous rhPTH(1-84) or rhPTH(1-34) via pump therapy during routine clinical care. We demonstrate that pump therapy is well tolerated and may have resulted in improved key parameters of mineral metabolism in most of the studied patients. These include normalization of serum calcium concentration and urinary calcium excretion as well as decreased serum phosphate concentrations. While our data does not show a lowering of the frequency of hypocalcemia in response to single daily PTH injections compared to conventional therapy, there was a decrease in the frequency of hypocalcemia in response to multiple daily PTH injections and PTH pump therapy. This difference may be attributable to the short half-life of rhPTH(1-84), which may not consistently maintain serum calcium levels when administered once daily, especially in this select group of susceptible patients who remained symptomatic and eventually required transitioning to PTH pump therapy.

Although we do not have information from standardized tools for the measurement of quality of life, pump therapy may improve symptoms and well-being in these patients, as indicated by a decrease in self-reported symptoms of hypocalcemia in all patients and less frequent emergency department visits observed in the 2 patients who had emergency room visits. On the other hand, episodes of pod failure were reported in 3 of 12 patients. This highlights the importance of planning for back-up therapies (ie, administration of PTH subcutaneous injections or oral calcium and active vitamin D or having a new pod and medication available) to prevent hypocalcemia in case of pod failure.

The efficacy of continuous subcutaneous delivery of hPTH(1-34) for treatment of hypoparathyroidism has been previously studied. In a 6-month randomized crossover study by Winer et al in 8 adult patients with postsurgical hypoparathyroidism, pump delivery of synthetic hPTH(1-34), compared with twice-daily injections, resulted in less fluctuation in serum calcium concentrations, over 50% reduction in urinary calcium, 65% reduction in the PTH dose to maintain normal calcium concentration, and higher serum magnesium concentrations [22]. These results were supported by another 6-month randomized crossover study by the same group in 12 children with congenital hypoparathyroidism showing that continuous pump therapy of hPTH(1-34), compared with twice-daily injections, resulted in near normalization of serum calcium concentration [mean ± SD: 8.1 ± 0.2 (pump) vs 7.5 ± 0.1 (injection) mg/dL, *P* < .05] and normalization of urinary calcium excretion [207.2 ± 44.1 (pump) vs 267.3 ± 30.5  (injection) mg/24 hours/1.73 m^2^, *P* = .30] [12]. Additionally, case reports have demonstrated favorable longer-term outcomes of continuous delivery of rhPTH(1-34) with respect to improving biochemical parameters in 3 children with hypoparathyroidism [17], 4 adults with hypoparathyroidism [18, 19], and 6 children and 1 pregnant woman with autosomal dominant hypocalcemia type I [20, 21].

Although the literature does provide some support for pump therapy with rhPTH(1-34) as an effective treatment for hypoparathyroidism outside of clinical trials, we believe this to be one of the most extensive case series on PTH pump therapy and the first report on the effects of pump therapy with rhPTH(1-84) in patients with chronic hypoparathyroidism. Another strength of our study is the inclusion of a large number of patients with a long observation time on pump therapy [2.9 ± 1.8 years (range 0.2-6.7 years)]. Additionally, our report represents real-world experiences and outcomes in managing patients with hypoparathyroidism by transitioning from conventional therapy to single/multiple daily PTH injections and subsequent PTH pump treatment.

It is worth noting that serum magnesium concentrations tended to be higher while on PTH pump therapy in several patients. This observation aligns with the findings of a prior clinical trial showing an increase in serum magnesium concentrations in response to PTH pump therapy compared with twice-daily injections [22]. This is likely due to the physiological action of PTH to enhance renal magnesium reabsorption by stimulating the uptake of magnesium into the distal convoluted tubule [25].

Our study carries some limitations that should be acknowledged. First, this study is a case series by design, and the 3 participating centers did not have uniform protocols. For instance, available 24-hour urinary calcium data are limited. Clinical trials and real-world data with larger prospective cohorts are warranted to further investigate the efficacy of this promising mode of administration of PTH. Second, the decision to transition from 1 mode of therapy to another was based on the patients’ symptoms, biochemical parameters, and shared decision-making, rather than a prespecified protocol. This complicates the interpretation of the data. Third, we relied on self-reported symptoms and well-being, not a standardized tool for measurement of quality of life, and thus the quality-of-life data is limited. Finally, we included patients who received rhPTH(1-84) and/or rhPTH(1-34), and some patients were switched from 1 therapy to another. This has compromised the homogeneity of the study data. It should be noted that rhPTH(1-34) is shorter-acting than rhPTH(1-84) when given as a subcutaneous bolus but otherwise has the same effects on calcium and phosphate homeostasis [8, 16]. Therefore, the biochemical outcomes of continuously administered rhPTH(1-84) and rhPTH(1-34) are not expected to be clinically significantly different. This may be supported by our results suggesting that there were no differences in the biochemical data between patients receiving rhPTH(1-84) pump and those receiving rhPTH(1-34) pump, although the confidence of this interpretation is limited by the small sample size.

## Conclusion

Continuous subcutaneous delivery of rhPTH by pump was well tolerated and may improve key parameters of mineral metabolism in patients with chronic hypoparathyroidism. In addition, pump therapy may improve symptoms and quality of life in these patients. It is important to note that pump therapy is a complex treatment modality that requires technical proficiency and manual dexterity on the part of the patient. Additionally, it demands specialized expertise from healthcare providers. Clinical trials and real-life experiences with larger cohorts are needed to confirm the efficacy of this promising mode of administration of rhPTH.

## Disclosures

D.W. was on an advisory board for Ascendis Pharma. M.M. is consultant for and receives research funding from Takeda, Calcilytix, and Amolyt. N.E.C. is a speaker and consultant for Alexion Pharmaceuticals, a consultant for Extend Biosciences, and site PI for Shire Pharmaceuticals/Takeda. All other authors do not have any conflict of interest.

## Data Availability

Some or all datasets generated during and/or analyzed during the current study are not publicly available but are available from the corresponding author on reasonable request.
